# Screening for cardiovascular risk in the general population: The SPICES implementation survey

**DOI:** 10.3389/fmed.2022.1058090

**Published:** 2023-01-16

**Authors:** Delphine Le Goff, Gabriel Perraud, Paul Aujoulat, Jeremy Deriennic, Morgane Guillou, Marie Barais, Jean-Yves Le Reste

**Affiliations:** ^1^Department of General Practice, University of Western Brittany, Brest, France; ^2^ER 7479 SPURBO, University of Western Brittany, Brest, France

**Keywords:** primary prevention, cardiovascular diseases, mass screening, students health occupations, population characteristics, implementation science

## Abstract

**Background:**

In 2019, cardiovascular diseases (CVD) caused 32% of deaths worldwide. The SPICES survey involved five countries in an international primary CVD prevention implementation study in the general population. The French SPICES survey was implemented in the Centre Ouest Bretagne area (COB), which is a rural, economically deprived, medically underserved territory with high cardiovascular mortality. A CVD screening in the general population was needed to select the implementation population without overburdening family practitioner (FP) workforces. The efficacy and the replicability of such a screening were unknown. The aims of this study were to identify the characteristics of the individuals undergoing CVD risk assessment with the Non-Laboratory Interheart risk score (NL-IHRS), and to identify barriers and explore facilitators when screening the general population.

**Methods:**

An implementation study combining a cross-sectional descriptive study with qualitative interviews was undertaken. The NL-IHRS was completed by trained screeners selected from health students, pharmacists, nurses, and physiotherapists in the area with a dedicated e-tool in sport and cultural events and public places. After the screening, all screener groups were interviewed until theoretical saturation for each group. Thematic analysis was performed using double-blind coding.

**Results:**

In 5 months, 3,384 assessments were undertaken in 60 different places, mostly by health students. A total of 1,587, 1,309, and 488 individuals were at low, moderate, and high CVD risk. Stressed or depressed individuals were remarkably numerous (40.1 and 24.5% of the population, respectively). Forty-seven interviews were conducted. The main facilitators were willingness of the population, trust between screeners and the research team, and media publicity. The main barriers were lack of motivation of some screeners, some individuals at risk, some stakeholders and difficulties in handling the e-tool.

**Conclusion:**

The efficacy of CVD risk screening while using mostly health students was excellent and preserved the FP workforce. Replicability was highly feasible if research teams took great care to establish and maintain trust between screeners and researchers. The e-tools should be more user-friendly.

## 1. Introduction

Cardiovascular diseases (CVDs) are the leading cause of death worldwide. They caused 17.9 million deaths in 2019. Prevalence of atherosclerotic CVD deaths decreased in high-income countries from 1990 to 2010 to 140.2 per 100,000 inhabitants (1). The prevalence of CVD is increasing in low- and middle-income countries. CVDs affect younger and working aging populations in these countries compared to high-income countries. In Sub-Saharan countries, CVD prevalence was 233.4 per 100,000 inhabitants in 2010. CVD leads to high direct and indirect costs. In Europe, in 2066, health care costs are estimated to be EUR10.79 billion, and non-healthcare costs are estimated to be EUR192.5 billion (2). From 2011 to 2015, economic loss due to CVD was estimated at USD3.7 trillion in low- and middle-income countries, and health care costs were sparsely documented (3).

Effective tools to identify high CVD risk people are mainly represented by biological scores. However, these tools need laboratory tests and consequent utilization of overused health systems to assess them. Non-biological scores have been used to develop new screening strategies: implementable strategies for the general population in low- and middle-income countries and wide low-cost screening of the general population in high-income countries. The non-laboratory Interheart risk score (NL-IHRS) is one of these. It was created from the INTERHEART case-control study data (4). The NL-IHRS was externally validated in 2013 (5). The score explores ten predictors of cardiovascular risk, such as age and gender, parental history of coronary artery disease, diabetes, hypertension, smoking history including secondhand smoke exposure, abdominal obesity, level of physical activity, reported depression and stress, reported diet including salt consumption, fruit consumption, vegetable consumption, fried food, trans-fat consumption, and meat and poultry consumption. The NL-IHRS global correlation to CVD is *C*-statistic = 0.69 (95% confidence interval [CI] 0.68–0.70) and is similar to that for myocardial infarction and stroke (4).

The scaling-up packages of interventions for cardiovascular disease prevention in selected sites in Europe and sub-Saharan Africa (SPICES) project involved five countries in an international primary CVD prevention implementation study in the vulnerable general population. Addressing vulnerability was a condition to request a H2020 grant. SPICES was funded in 2017. High-income countries were represented by England, Belgium, and France which identified vulnerable populations in their own territory. Low- and middle-income countries were, respectively, represented by Uganda and South Africa. Vulnerable population was defined by consensus within the international research team as any economically deprived population, with low access to prevention and to care. The aims of the overall project were to tailor and develop CVD interventions that would be complementary to local current strategies and monitor implementation clues as acceptability, adoption, appropriateness, feasibility, fidelity, and costs. Each research team designed primary CVD prevention interventions adapted to their context after structured baseline assessments.

The complete French protocol for the SPICES implementation has been published (6). The first phase was a screening of CVD risk in the general population using a web-based NL-IHRS, which enabled to recruit participants at moderate CVD risk phase, and the description of this phase was the objective of this article. The second phase was the implementation of a community-based intervention to improve CVD risk. In France, screening is mainly delegated to family practitioners (FPs). Currently, FP access is becoming difficult as the number of FPs is declining. Local initiatives from mutual health insurance are ongoing, and global health checks are proposed for some of the salaried employees. However, these screening strategies are uncoordinated, targeted on specific individuals without involving the general population, and not efficacious (7).

The French SPICES survey was implemented in the Centre Ouest Bretagne (COB) area, which is a rural, economically deprived, medically underserved territory with high cardiovascular mortality. There was excess mortality of 30% among men and 19% among women compared to the mean French mortality. In 2010, the COB territory had an estimated 8.9 FPs and 1.2 specialized physicians per 10,000 inhabitants compared to the mean of 13.1 and 17.2, respectively, in France (8).

A screening strategy to identify individuals with high CVD risk without overwhelming FPs was built for the French SPICES phase 1. In 2018, a new mandatory preventative health internship of 6 weeks was created in France for health students. The French SPICES phase 1 screening was in the scope of its program. A total of 280 health students were recruited for the intervention (9).

The conjunction of a non-biological validated score for CVD risk assessment and the creation of the French preventative health internship provided an innovative framework for CVD risk assessment screening strategy in the general population. The French part of SPICES was designed as an implementation hybrid type 1 study to explore barriers and facilitators at each step of the survey for a better understanding of quantitative results (10). The aims of the French SPICES phase 1 were first to find intermediate CVD risk individuals in the general population, then describe the characteristics of the created cohort following the NL-IHRS for the intervention phase, and finally, collect and classify the barriers and facilitators to screen the general population.

## 2. Materials and methods

An implementation hybrid type 1 study combining a cross-sectional descriptive study with qualitative interviews was undertaken. This study was reported following the standard for reporting implementation studies (StarI) statements and the Consolidated framework for implementation research (CFIR) (11, 12).

### 2.1. Setting recruitment

A standard procedure was followed to get access to local festive events and medico-social organizations. First, the Community Health Project Manager identified a referent in each town of the COB area and obtained a list of the events and organizations likely to welcome screeners from that referent. Then, the research team contacted the manager of each event or organization to request an invitation and agreed on dates, times, and the number of persons expected at the event. The research team then estimated the number of screeners needed for each event. All invitations were collected and placed on a global schedule allowing the research team to allocate a junior researcher and an adequate number of screeners to each event. Other voluntary medical organizations or professionals were integrated into the screening phase at the request of the research team.

### 2.2. Screeners recruitment and training

The SPICES screening complied with the preventative health service decree (9). Accordingly, the Faculty of Medicine, the physiotherapy school and the nursing school required their students to participate. A total of 280 students who had to perform their preventative health internship were enrolled in the screening. They were trained by a 1-day-long session about using the dedicated tablets, communication skills, delivering the NL-IHRS and the brief advice, ethics of research, and the anonymization procedure. Junior researchers followed the same curriculum plus training on screeners’ guidance and data transfer. The training was later adapted to COB professionals involved on their request: pharmacists, nurses, physicians, desk team trainees of the outpatient hospital. For the last public events, volunteer FP interns who were required to perform a research project were trained to assume latest screening invitations.

### 2.3. NL-IHRS implementation on web-based tools

A blinded translation of the NL-IHRS was undertaken by two researchers, and a consensus meeting with a third researcher was conducted to produce a French version of the NL-IHRS. A template was created on the REDCap software^®^ (Vanderbilt University, Nashville, USA) according to the data protection regulations. A search in the French guidelines was conducted for each CVD risk factor explored by the NL-IHRS to create a standardized, appropriate brief advice. Brief advice adapted to stress and depression was created and validated by the local scientific committee as no specific recommendation existed. The committee comprised addiction specialists, psychiatrists, and FPs.

Each piece of generated advice was standard but only appeared on the tablet when it fitted the participant’s answer. REDCap^®^ securely hosted the data.

### 2.4. Deployment of screeners

During the preventative health internship, teams of five to six screeners were created. For every 50 people expected in an event, a team, dressed in SPICES windbreakers, was moved to the event. According to the number of teams displaced, 1–10 junior researchers accompanied the screeners on the field. The COB professionals, pharmacists, nurses, physiotherapists, and desk team trainees of the outpatient hospital offered screening during their care routine. After the intervention of the preventative health internship ended, new invitations of COB stakeholders arrived to the research team. These stakeholders were attracted by the feedback of the first screening experiences on the field. FP interns were trained to respond to these invitations and performed screenings. In the field, screeners canvassed participants, assessed the NL-IHRS, and delivered the appropriate brief advice. When they screened someone at intermediate cardiovascular risk, they offered the participant inclusion in the second phase of SPICES. Participants willing to be included gave their identity in a separate sheet collected by the junior researchers. Participants at low risk were given brief advice and positive reinforcement. Participants at high risk were given brief advice and were strongly recommended to get an appointment with their usual physician.

### 2.5. Eligibility criteria

Participants belonged to the general population. They were aged >18 years and worked or lived in the COB. Non-inclusion criteria for the people undergoing the screening were: age <18 years, current pregnancy, living and working outside COB, and personal history of CVD.

### 2.6. Variables

Clinical data collected were the results of the NL-IRS (5). The NL-IRS comprised 10 scored items. The first item combined age and gender, the second item explored parental history of coronary artery disease, the third, self-declared diabetes, the fourth, self-declared hypertension, the fifth, smoking history, the sixth second-hand smoke exposure, the seventh, level of physical activity, the eight, psychosocial factors including reported depression and reported general stress, the ninth, reported diet including salt consumption, fruit consumption, vegetable consumption, fried food, trans-fat consumption, and meat and poultry consumption. The tenth predictor was the waist-to-hip ratio. The score was ranked from 0 to 48. The score categorized the population into three groups depending on their CDV risk: low risk if the NL-IHRS was <9, moderate risk if the NL-IHRS was between 9–15, and high risk if the NL-IHRS was >15.

The administrative data collected from screening participants were full name, phone, and e-mail. These data were recorded separately from the results of the NL-IHRS, accordingly to the ethics board recommendations. Informed consent was required participating. Categorization of screening sites used definitions of the French *institut national de la statistique et des études économiques* (INSEE) (13), especially for the distinction between rural and urban areas.

### 2.7. Resource use, costs, and economic outcomes

The costs of the project were borne by two entities. The European Union grant founded expenditures on equipment comprising SPICES windbreakers for every screener, tablets, tape measures, consent forms for every participant, recording sheets for the second SPICES phase, posters for screening sites. The grant funded a full-time clinical research associate during the screening. The grant also reimbursed mileage expenses of screeners for their travels from the faculty to the screening sites. Researchers and FP interns received their current salary from the French state, students received a state lump sum compensation for the completion of their preventative health service also from the French state. The field professionals were unpaid volunteers.

### 2.8. Data sources/Measurement

Nine items of NL-IHRS were declarative items. The only measurement was the waist-to-hip ratio, which was measured by the screeners according to the training. Measurement quality was enhanced by the junior researcher’s supervision of screeners.

### 2.9. Biases

Information biases were handled using a standardized tool, the REDCap-based NL-IHRS, and the training of both screeners and junior researchers. Selection biases were limited by targeting different events and organizations to sample different populations.

### 2.10. Study size

The number of events and organizations was modified until the research team could recruit 1,000 participants at intermediate cardiovascular risk for the second SPICES phase. The protocol of the French SPICES survey has been previously published (6). The second SPICES phase tested a community behavioral intervention to reduce CVD risk. The sample size of 1,000 participants was calculated to show a 15% difference on the NL-IHRS after a 24-month intervention.

### 2.11. Statistical methods

Descriptive epidemiology was used to describe the population. The population was first described as a whole using the NL-IHRS (Table 1) and then by the three risk categories. Comparisons were performed between males and females, participants older than 65 years and younger, and rural and urban inhabitants. Screening settings were inductively categorized into 11 categories: sport events, cultural events, retiree events, charitable events, workplaces, sheltered workplaces, local and regional governments, medical facilities, markets and supermarkets, paramedics, and pharmacies. The distribution of the population was described by categories. Comparisons from an implementation perspective were performed between workplaces screening and others, sheltered workplaces and others, pharmacies and others, paramedics and others, sport events and others, administrations and others, and preventative health internship recruitment and others. For each comparison, records were specifically excluded if they had missing data compromising the comparison. Comparisons were made using Student tests for quantitative variables of the NL-IHRS, and Chi^2^ tests were used for qualitative variables of the NL-IHRS. Differences were statistically significant if *p* was <0.05. Analyses were performed using SAS, version 9.4 (SAS Institute, Inc., Cary, NC, USA).

**TABLE 1:** Features of the 3,384 SPICES cohort subjects.

Variable		Total (*N* = 3,789)
Age	N (missing)	3,458 (331 missing)
	Mean ± Standard deviation	54.30 (16.26)
	Median (q1; q3)	57.0 (43.0; 67.0)
	Min; Max	16; 96
Age group	N (missing)	3,458 (331 missing)
	Aged 65 or under	2,501 (72.3%)
	Aged over 65	957 (27.7%)
Gender	N (missing)	3,498 (291 missing)
	Male	1,308 (37.4%)
	Female	2,190 (63.6%)
Living area	N (missing)	3,482 (307 missing)
	Rural	1,728 (49.6%)
	Urban	1,754 (50.4%)
Screening location	N (missing)	3,413 (376 missing)
	Sport events	901 (26.4%)
	Cultural events	254 (7.4%)
	Retiree events	71 (2.1%)
	Charitable events	226 (6.6%)
	Workplaces	102 (3%)
	Sheltered workplaces	146 (4.3%)
	Administrations	122 (3.6%)
	Medical facilities	710 (20.8%)
	Markets and supermarkets	98 (2.9%)
	Paramedics	143 (4.2%)
	Pharmacies	640 (18.8%)
Screener	N (missing)	3,514 (275 missing)
	Others	579 (16.5%)
	Preventative health internship	2,935 (83.5%)
**Interheart risk score**		
Smoking	N (missing)	3,491 (298 missing)
	I never smoked (%)	1,855 (53.1%)
	I am a current smoker 1–5 cig/day (%)	198 (5.7%)
	I am a current smoker 6–10 cig/day (%)	164 (4.7%)
	I am a current smoker 11–15 cig/day (%)	147 (4.2%)
	I am a current smoker 16–20 cig/day (%)	84 (2.4%)
	I am a current smoker >20 cig/day (%)	65 (1.9%)
	I am a former smoker (last smoked more than 12 months ago) (%)	978 (28.0%)
Secondhand smoke	N (missing)	3,424 (365 missing)
	Less than 1 h or exposure per week or no exposure (%)	2,822 (82.4%)
	One or more hours of second-hand smoke exposure per week (%)	602 (17.6%)
Diabetes mellitus	N (missing)	3,424 (365 missing)
	No or unsure (%)	3,279 (95.8%)
	Yes (%)	145 (4.2%)
High blood pressure	N (missing)	3,424 (365)
	No or unsure (%)	2,779 (81.2%)
	Yes (%)	645 (18.8%)
Family history	N (missing)	3,424 (365)
	No or unsure (%)	2,820 (82.4%)
	Yes (%)	604 (17.6%)
How often have you felt work or home life stress in the last year?	N (missing)	3,425 (364)
	Never or some periods (%)	2,053 (59.9%)
	Several periods or permanent stress (%)	1,372 (40.1%)
During the past 12 months, was there ever a time when you felt sad, blue, or depressed for 2 weeks or more in a row?	N (missing)	3,425 (364)
	No (%)	2,586 (75.5%)
	Yes (%)	839 (24.5%)
Do you eat salty food or snacks one or more times a day?	N (missing)	3,425 (364 missing)
	No (%)	3,238 (94.5%)
	Yes (%)	187 (5.5%)
Do you eat deep fried foods or snacks or fast foods 3 or more times a week?	N (missing)	3,423 (366 missing)
	No (%)	3,151 (92.1%)
	Yes (%)	272 (7.9%)
Do you eat fruit one or more times daily?	N (missing)	3,424 (365 missing)
	No (%)	730 (21.3%)
	Yes (%)	2,694 (78.7%)
Do you eat vegetables one or more times daily?	N (missing)	3,424 (365 missing)
	No	491 (14.3%)
	Yes	2,933 (85.7%)
Do you eat meat and/or poultry 2 or more times daily?	N (missing)	3,425 (364 missing)
	No (%)	2,685 (78.4%)
	Yes (%)	740 (21.6%)
Physical activity	N (missing)	3,424 (365 missing)
	I perform moderate or strenuous physical activity in my leisure time (%)	2,496 (72.9%)
	I am mainly sedentary or perform mild exercise (requiring minimal effort) (%)	928 (27.1%)
Waist to hip ratio	N (missing)	3,398 (391 missing)
	Mean ± Standard Deviation	0.91 (0.08)
	Median (q1; q3)	0.9 (0.9; 1.0)
	Min-Max	0; 2
NL-IHRS	N (missing)	3,384 (405 missing)
	Mean ± Standard Deviation	9.71 (5.60)
	Median (q1; q3)	9.0 (6.0; 13.0)
	Min-Max	0; 34
NL-IHRS category	N (missing)	3,384 (405 missing)
	Low risk (%)	1,587 (46.9%)
	Intermediate risk (%)	1,309 (38.7%)
	High risk (%)	488 (14.4%)

### 2.12. Qualitative data

Qualitative, individual, semi-structured interviews were performed to capture barriers and facilitators related to screening. Qualitative purposive samplings of screeners were created among preventative health internship students, pharmacists, paramedics, FP interns, and members of the research team. Sampling criteria included the role in the screening, age, gender, location of the screening for every group. A brainstorming was organized within the research team to identify particular individual characteristics among screeners which could specifically diversify answers in the interviews. The interviews were audio recorded. They were then after transcribed into verbatim quotes and de-identified. Records were destroyed after transcription. Data collection and analysis were iterative, so that early data analysis influenced further interviews (14). Interview guides were consequently adapted along with the interviews. Interviews were performed within each group until theoretical data saturation. A thematic analysis was performed by two researchers working blind, coding the data, and eventually merging their analyzes. Themes were consecutively integrated into a CFIR construct. The CFIR template was adapted. The original numbering scheme of the CFIR template was maintained to allow further comparisons.

## 3. Results

Screening with the preventative health internship was planned from April to early July 2019. The screening was extended with the local workforce and FP trainees until September at the request of local organizations and stakeholders. In 5 months, 3,384 assessments were undertaken in 60 different places. Finally, 1,309 people at intermediate CVD risk were found.

### 3.1. General overview of the screening

A total of 1,309 people had intermediate CVD risk following the NL-IHRS, 1,587 people had low CVD risk, and 488 people had high CVD risk. Mean NL-IHRS was 9.71 (±5.60). The cohort included 1,308 men and 2,190 women. Of all, 40.1% of people declared being stressed, and 24.5% of people declared being depressed. Additionally, 18.8% of people were current smokers, and 27.1% of people were sedentary. Global features of the cohort are presented in Table 1.

### 3.2. Screening features by settings

Among the 60 recruited sites, 10 were sport events, 5 were cultural events, 2 were retiree events, 4 were charitable events, 3 were workplaces, 3 were sheltered workplaces, 4 were administrations, 10 were medical facilities (visitors and non-cardiologic outpatients), 1 was a supermarket, 1 was a marketplace, and 16 were pharmacies, nurses and physiotherapists were considered as one entity.

**FIGURE 1:**
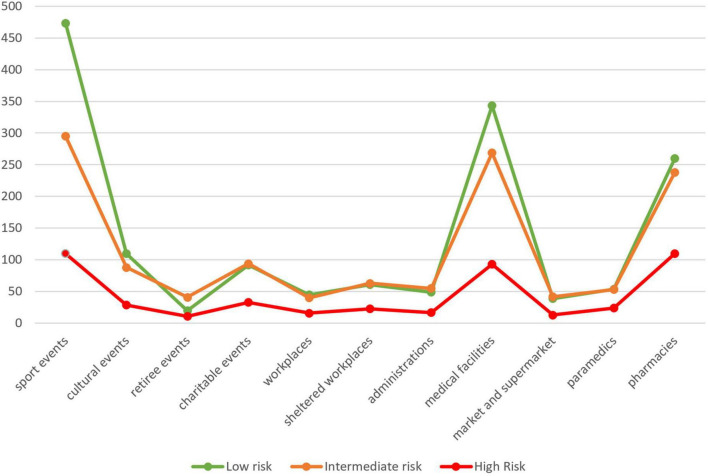
Number of people screened by NL-IHRS categories and settings.

Among the settings, number of screenings varied, but populations met were also different. More screening was conducted in sport events (*n* = 878), but the percentage of people at low CVD risk was high (53.9%). In comparison, screening in pharmacies was less extensive (*n* = 608) but recruited more people at intermediate or high risk (low risk: 42.8%). Retiree events had a higher rate of intermediate- and high-risk people because of age and history of diabetes and hypertension. Number of screenings and percentages of risk categories are presented in Figure 1.

### 3.3. Stress and depression

**FIGURE 2:**
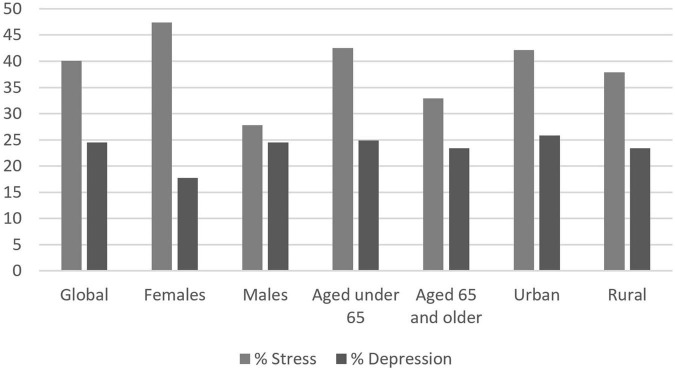
Declared levels of stress and depression.

Among the whole cohort, 40.1% of people declared being stressed, and 24.5% of people declared being depressed, which was unexpected. Compared to men, women were significantly more stressed (47.4 vs. 27.8%, *p* < 0.001) and more depressed (28.7 vs. 17.7%, *p* < 0.001). Younger people were significantly more stressed than older individuals, but depression was equally declared in both groups. Urban residents were significatively more stressed than rural residents (42.1 vs. 37.9%, *p* = 0.013) but equally depressed. Percentages of declared stress and depression are presented in Figure 2.

### 3.4. Qualitative analysis of barriers and facilitators

A total of 47 interviews were conducted. Mean duration of the interviews was 26 min, varying from 5 to 71 min. The interview guide was independently modified for each group from one to four times (final guides are presented in Supplementary material). In each group, interviews were conducted until data saturation except for the FP interns’ group. This group comprised seven people that were all interviewed. Sampling characteristics are presented in Table 2. In addition to age, gender and location of the screening, specific characteristics were searched as they were presumed to influence answers. For the research team, working inside the COB area or in the distant metropolis was sought to be influencing. In the same way, to stop participating to the project was a selection criterion. For the preventative health service, students were relatively similar regarding age, medical background and studied in the same faculty. It was thought that students from rural or semi-rural could have a different perception of the COB area than urban students. For paramedics who worked in similar structures, a geographical diversification was retained as the COB area spans three departments with different governances. Professional organizations of pharmacists could be very different with big structures employing many professionals, so this criterion was retained for diversification. Furthermore, it was sought that a solitary participation within a pharmacy would lead to different answers than a collective experience. The main difference for FP interns was their progress in their curse, older FP interns being more experienced and focused on their future professional activity. Main facilitators were readiness and involvement of stakeholders, population, and health care professionals, trust between screeners and research team, and media spread. Main barriers were the lack of motivation and difficulties to handle the e-tool. Facilitators, barriers, and neutral factors were classified in the CFIR template, as presented in Table 3.

**TABLE 2:** Sampling characteristics of the qualitative interviews.

Group	Ref	Gender	Age	Position	Place of work	Additional information
Research team	RT1	F	40	Practicing physician	Brest	Currently involved
	RT2	F	61	Stakeholder, champion	COB country	Currently involved
	RT3	F	31	Practicing physician	Brest	Participation discontinued
	RT4	F	39	Clinical research associate	Brest	Currently involved
	RT5	F	25	Stakeholder	COB country	Currently involved
	RT6	F	35	Financial reporter	Brest	Currently involved
	RT7	F	40	Clinical research associate	Brest	Participation discontinued
	RT8	M	33	Practicing physician	Brest	Participation discontinued
	RT9	M	73	Stakeholder	COB country	Currently involved
	RT10	M	28	Master student	Brest	Currently involved
	RT11	M	65	Practicing physician	Brest	Currently involved
Preventative health service	PHI1	M	22	Medical student	Brest	Semi-rural origin
	PHI2	F	23	Nurse student	Brest	Urban origin
	PHI3	F	20	Physiotherapy student	Brest	Semi-rural origin
	PHI4	F	22	Medical student	Brest	Semi-rural origin
	PHI5	F	21	Physiotherapy student	Brest	Semi-rural origin
	PHI6	F	21	Medical student	Brest	Semi-rural origin
	PHI7	F	23	Medical student	Brest	Urban origin
	PHI8	F	20	Nurse student	Brest	Urban origin
	PHI9	F	24	Medical student	Brest	Rural origin
	PHI10	F	19	Nurse student	Brest	Rural origin
Paramedics	PM1	F	39	Physiotherapist	Finistère, rural	
	PM2	M	40	Private practice nurse	Morbihan, rural	
	PM3	F	37	Private practice nurse	Morbihan, rural	
	PM4	F	61	Physiotherapist	Finistère, urban	
	PM5	F	34	Private practice nurse	Finistère, urban	
	PM6	M	46	Physiotherapist	Finistère, rural	
	PM7	F	41	Private practice nurse	Finistère, rural	
	PM8	F	63	Private practice nurse	Finistère, rural	
	PM9	F	64	Physiotherapist	Finistère, rural	
Pharmacies	Ph1	F	44	Pharmacist	Finistère, rural	3 members team, 3 screeners
	Ph2	F	44	Pharmacy assistant	Morbihan, rural	8 members team, 4 screeners
	Ph3	F	38	Pharmacist	Morbihan, rural	9 members team, no screener
	Ph4	F	34	Pharmacy assistant	Finistère, semi-rural	6 members team, 2 screeners
	Ph5	F	47	Pharmacist	Finistère, rural	3 members team, 3 screeners
	Ph6	F	45	Pharmacy assistant	Côtes d’Armor, rural	4 members team, 4 screeners
	Ph7	F	34	Pharmacist	Côtes d’Armor, rural	7 members team, 2 screeners
	Ph8	F	37	Pharmacy assistant	Côtes d’Armor, rural	6 members team, 2 screeners
	Ph9	F	35	Pharmacist	Côtes d’Armor, rural	2 members team, 1 screener
	Ph10	F	30	Pharmacy assistant	Côtes d’Armor, rural	8 members team, 4 screeners
FP interns	FPI1	M	28	FP intern	Brest	Third year of internship
	FPI2	F	26	FP intern	Brest	Third year of internship
	FPI3	F	28	FP intern	Brest	Third year of internship
	FPI4	F	33	FP intern	Brest	First year of internship
	FPI5	M	28	FP intern	Brest	Third year of internship
	FPI6	M	29	FP intern	Brest	Second year of internship
	FPI7	M	27	FP intern	Brest	Second year of internship

**TABLE 3:** Facilitators, barriers, and neutral factors summarized following CFIR constructs.

Domain and construct				
**I. Intervention characteristics**				
A	Intervention source		Facilitators	The overall intervention and the screening were externally designed by the research team but adapted with the local stakeholders.
			Barriers	A barrier for internal development was the distance between the premises of the research team and the COB area.
B	Evidence strength and quality		Facilitators	Stakeholders perceived the project as innovative, carrying human values. The project was complementary of other preventative health actions launched in the territory. An intervention on cardiovascular risk was deemed appropriate to health problems of inhabitants.
			Barriers	Despite the research team communications, some stakeholders remained unfamiliar with the project or disinterested.
C	Relative advantage		Facilitators	The project was perceived as addressing the lack of prevention in France. Cardiovascular prevention fit to the populations’ health. Stakeholders expressed that hosting the project improved the image of the COB territory. The deployment of screeners and the publicity around the project valorized the COB territory. The screening was deemed acceptable by the screeners. The screening was perceived as an introduction to further deeper preventive actions. The population was receptive to the SPICES project and adhered to screening. Participating in the project led the health students to discover the COB territory.
			Barriers	Some screeners described preventive professional skills as something new in their practice. Screeners felt announcing a high cardiovascular risk was challenging. Finally, some participants had requests beyond the screening and brief advice that could lead to discomfort for screeners.
D	Adaptability		Facilitators	During the events, some screeners organized spontaneously a new position of canvasser which referred potential participants to screeners and increased participation to the screening. For health professionals, shifts in screening, use of waiting time in queues at pharmacies, creation of dedicated times were innovations to perform the screening. Some nurses integrated screening in their routine care. In local events, organizers were facilitators by placing signs, setting up a booth and making announcements on the microphone. At times, screeners used the consent form within groups to promote screening. Some screeners printed a paper version of the NL-IHRS to deal with the tablet remotely.
			Barriers	Barriers to adaptability were shortness of the recruitment period for health professionals, and rigidity of European funding which complicated the purchase of equipment, the compensations for screeners. The rigidity of the European financial lines prevented reallocations while the research team refined study needs. Calendar constraints frustrated preventative health service students, encroaching on weekends, holidays, summer jobs. The geographic exclusion criterion for participants was annoying according to preventative health service screeners as foreigners to the COB area participated to COB events and were disappointed that they could not participate.
F	Complexity		Facilitators	Supervision of screeners by junior researchers was perceived as a strength for facing complexity as they could solve tablet problems, communication issues, personal health problems brought by participants. Screeners used social networks to facilitate deployment of screeners. The NL-IHRS was perceived by screeners as representative of cardiovascular risk.
			Barriers	Unexpectedly, screeners discovered that the population was redundant from an event to the next. For health professional, recruiting during summer was arduous because of colleagues’ vacations inducing extra-work. Some screeners feared biases in the NL-IHRS because of embellishment of answers by participants and the absence of questions about alcohol.
			Neutrals	Duration of the training, content of the training, handling of the tablets during the screening, recruitment duration and some considerations about the NL-IHRS (classification of the answers, feasibility of the measure) were considered either barriers or facilitators.
G	Design quality and packaging		Facilitators	The NL-IHRS and the brief advice were easy and short to deliver. The brief automated advice gave meaning to the NL-IHRS for participants. Using a tablet was acceptable for participants. The auxiliary material was small enough to allow screeners autonomy and ambulation in events. The SPICES windbreakers made screeners visible and attracted people to the screeners. Wording of the NL-IHRS questions was clear. Posters created by the research team were effective in attractiveness. Some events had specific signaling, even a specific booth or room dedicated to the screening which improved attractiveness of screening.
			Barriers	When walking around, it was difficult to handle simultaneously the tablet sleeve, the tablet, and the tape measure. Due to lack of supply, windbreakers were navy-blue instead of red, which reduced visibility of the screeners. Regulatory content in the consent form made the consent form overly complex to understand for participants. Several tablet bugs were encountered: touch screen malfunction, tablet failures, random switch of software from French to English. Tablets were new supports for patients. Tablets could disrupt interactions between screeners and participants. There was a need for spare tablets. The software was found to be unintuitive with a long connection delay. The Redcap application was judged as poorly coded with imprecise wording of application menus. Data transfer suffered from the lack of acknowledgment of receipt of the data, low internet speed and a difficulty to handle data transfer procedure. Some screeners regretted the absence of pictures to illustrate the NL-IHRS questions.
H	Cost		Facilitators	The European funding was perceived as a strength as the COB stakeholders did not have to clear a budget to deploy the screening.
			Barriers	Some costs were not anticipated, as a financial compensation for COB structures which were involved in publicizing the project in the COB area.
**II. Outer setting**				
A	Needs and resources of those served by the organization		Facilitators	Many screeners perceived this was their role to address health prevention. They described a professional consistency in being engaged as screeners. Participants declared a particular interest in their health. Relatives could press participants for screening. Participants shared their knowledge in cardiovascular health with screeners. Participants expressed they were looking for solutions to improve their health.
			Barriers	Screeners felt there was not public demand for screening in events. When screening in companies, the screening was in competition with working time or break time. Due to the very low medical density of the territory, some participants had no doctor to refer and to handle elevated NL-IHRS result.
B	Cosmopolitanism		Facilitators	Health professional screeners expressed a sense of belonging to the community. Participating to the screening provoked a federation of the professionals around the project. For health professionals, the pre-existing relationship was a facilitator to propose the screening.
			Barriers	Screeners perceived some events were not suitable for screening, for example a community garage-sale. An organizer tried to hijack the screeners to perform first aid in his event. In some pharmacies, partial involvement of the team was a barrier to perform screenings in large numbers.
**III. Inner setting**				
A	Structural characteristics		Facilitators	Events took place in a good atmosphere; screeners received a warm welcome. Some specific logistics in events were particularly suitable as visible layout in the event, dedicated room for screening, prior internal promotion to the screening, dedicated oral announcements in the event and hierarchical incentive for screening in some companies.
			Barriers	Some failures in the organization of the events themselves had repercussions on screening: signage of the event itself, signage of the screening in the event, late promotion of the screening in the event, lack of electricity, lack of privacy. Large events were difficult to canvass for screeners. Movement of people in some events prevented screeners to catch participants. Some screeners felt populations were selected according to the theme of the events. Some events suffered unexpected low attendance.
B	Networks and communications		Facilitators	The research team, the stakeholders and the screeners used diversified means of communication as text messages, WhatsApp, Google drive, physical meetings. The research team promoted the project using paper media and radio. Screeners described a group dynamic among themselves. They planned carpooling to events. Screeners pointed out effective support from supervising junior researchers. Screeners identified team spirit, mutual aid, and emulation in the group. Screeners organized peripheral convivial moments, they described bonding together. Good relations existed within and with the research team. Research team members knew each other well. The research team was available to the stakeholders and the screeners. Junior researchers appreciated their back-up groups that got them support and enhanced their work.
			Barriers	In some events, screeners did not organize themselves, and distributed no dedicated roles leading to relative inefficiency.
C	Culture		Facilitators	Screening was felt by screeners like the continuity of usual talks between health professionals and patients. Many screeners related this experience with previous trainings in cardiovascular prevention. The topic of cardiovascular prevention was already an interest of screeners. Screeners felt they had a role in health promotion. They felt they created a possibility to continue prevention beyond brief advice.
			Barriers	None
D	Implementation climate		Facilitators	The screening was strongly welcomed by local stakeholders, local associations, and screeners. Screening plus brief advice was perceived as a human sharing. Trust was a value commonly shared within the study: between health students and junior researchers, between patients and health professionals, within the research team and between local actors and the research team.
			Barriers	Preventative health service students took the training in a bad mood because of the encroachment of the project on their schedules. Some screeners had non-professional behaviors: absence to the training, absence to the screenings, lateness to events, hangovers, and alcoholism. Some participants rejected the screeners by mentioning an inappropriate expertise, a difference in social class, conspiracy, and lassitude. Some participants expressed bad emotions. Some screeners expressed doubts about the interest of the study. The risk announcement could be badly experienced, participants could be disappointed by exclusion and reassurance for an unexpected score could be difficult. Some participants were not paying attention to the screening. Some people got aggressive talking about low medical demography. Participants could feel an intrusion with the NL-IHRS or initiate off-topic discussions.
	1	Tension for change	Facilitators	Screening was perceived by screeners as an opportunity to listen to participants. The screening was experienced as a reward for both the screener and the participant. Participants were curious about the assessment. Participants expressed a benefit to get a contact with a caregiver. Screeners and participants were interested in participating to a clinical study. Some participants were searching for the follow-up of the second SPICES phase. Screeners expressed a pleasure in carrying out the screening.
			Barriers	As an unusual task, health professionals could forget to offer the screening. In events, screening was an unusual proposal and participants could express reluctance to be canvassed. Screeners felt excessive expectations from some people local to the area. Population could have in contrast a lack of interest about cardiovascular prevention or a lack of motivation to improve their health. Some participants argued they already had a follow-up or had competing priorities to cardiovascular health for not carrying out the screening.
	2	Compatibility	Facilitators	The screening was a continuity of usual conversations. The topic of cardiovascular prevention was already an interest of screeners. Many screeners perceived this was their role to address health prevention.
			Barriers	However, screeners underlined the lack of institutional recognition of prevention and the lack of financial valorization of prevention. Medical students underlined their lack of awareness of prevention entailed by their current training.
	3	Relative priority	Facilitators	None
			Barriers	Expressed barriers were the competitive professional priorities for health professionals, competitive personal priorities for professionals, students, and research team.
			Neutrals	Time to allocate to the screening and current health professionals’ workload were perceived either as facilitators or barriers.
	5	Goals and feedback	Facilitators	None
			Barriers	Thinking about recruitment goals frightened some screeners as they felt they could not fulfill this objective. Preventative health service students deplored the lack of feedback of screening results from the research team. Imprecisions in the grant protocol hindered a clear communication of goals to stakeholders and led to an initial blurred communication from the research team.
	6	Learning climate	Facilitators	Screeners described a progressive empowerment during the screening. Being involved in the project resulted in a gain of knowledge, skills, and legitimacy for the screeners. Gradually, screeners expressed a familiarity with the NL-IHRS. They gained confidence in screening and expressed a progressive empowerment. Screeners developed recruitment strategies as the creation of the canvasser, effective presentation speech, search for areas of affluence, taking advantage of a snowball effect for attracting participants, the targeting of groups in events, a splitting of screeners, some staying in the booths and some wandering. The research team expressed an important collaboration within it. A specific recruitment was realized to focus on organization tasks in the research team (research internship plus secretary). Collaboration between junior researchers and the research team was appreciated on both sides. Consultation of local actors by the research team was appreciated by local stakeholders.
			Barriers	The research team was frequently on a rush with precipitations in the organization. In screening groups, some mutual unfamiliarity of the members could be uncomfortable. Screeners expressed some dissatisfaction because of the geographical remoteness and the obligation to participate. From the participant’s point of view, facing a group of screeners could generate a feeling of oppression.
E	Readiness for implementation		Facilitators	Health professionals volunteered to screen. Screeners expressed voluntarism in recruiting people and screening people. Local associations, work supervisors and families encouraged participants to perform the screening. The welcome on the events was benevolent with dedicated announcements and dedicated booths. Health professionals, declared a legitimacy in the screening and pharmacies were especially accessible for participants.
			Barriers	The participation of the screeners to a single event was forgotten by the organizers of the event despite reminders of the research team.
	1	Leadership engagement	Facilitators	The research team members were very available and deeply involved in the study. Human qualities of the doctors involved in the research team were esteemed. The research team engaged in regular communication with local stakeholders. The research team had respect for the privacy of team members. Research staff described less stress than hospital projects. In the COB, there was a local attractiveness of SPICES.
			Barriers	There was a competition in the researchers’ agenda and an overflow on researchers’ personal time.
	2	Available resources	Facilitators	The specific resources available for the study which were: - Two specific recruitments in the research team including a student for his research internship and a secretary - Junior researchers - Screeners - Tablets - The Redcap application and its remote data backup - The automated brief advice was efficient. - The consent form and the small auxiliary material - Lent equipment for screeners by events’ organizers - SPICES flocked windbreaker - Posters - A paper version of the NL-IHRS was added by some screeners
			Barriers	Researchers and screeners complained about the lack of research staff, no specific premises in some events, a lack of booths or shelters against the rain, a clutter by personal belongings, an absence of a standard rationale, the overly complex consent form, and the absence of a printed questionnaire.
	3	Access to knowledge and information	Facilitators	The training was appreciated by screeners for its presentation of the project, the peer training, the group training and the NL-IHRS self-scoring during the training.
			Barriers	However, screeners regretted that the training did not mention more on the expectations of the population of the COB area, methods for canvassing, deeper experience of the NL-IHRS, cues for good relationship with participants. Some screeners needed more precisions about the objective of the study. Some screeners found that trainers had excessive assumptions about their digital skills. For the first trainings, the definitive version of the French NL-IHRS was not available. Screeners had new needs which appeared between training and screening and had new technical needs when they manipulated the tablets, these needs were not covered by the training.
			Neutrals	Cardiovascular knowledge provided by the training, duration of the training and the handling of the tablets were either considered as facilitators or barriers.
**IV. Characteristics of individuals**				
A	Knowledge and beliefs about the innovation		Facilitators	The screening was attractive because screeners could be part of a research project. Screeners felt they mastered the topic, and it was reassuring for patients to face a health professional for such a screening. Junior researchers expressed belonging to the SPICES project was rewarding.
			Barriers	On the other side, some professional inconsistency could arise as some screeners felt their professional skills were limited to handle cardiovascular screening. For preventative health students, the screening appeared too early in their training course. Some screeners felt incompetent in canvassing. Screeners and junior researchers were inexperienced. Some screeners had no digital skills.
B	Self-efficacy		Facilitators	Screeners described themselves as having a quality of contact. They were able to adapt themselves to the participant’s personality and to use humor wisely.
			Barriers	On the other side, some screeners considered they lacked self-confidence, and motivation. Some screeners were shy. Some screeners had fears of refusal, fear of facing the participants’ answer, fear of announcing the results, fear of breaking the equipment, fear of filling error, fear of being intrusive. Communication could be difficult, some screeners lacked clarity, made offensive formulations, experienced discomfort in facing people at risk, had difficulties in popularizing medical information. Accumulation of refusals and repetition of tiresome screenings led some screeners to discouragement.
C	Individual stage of change		Facilitators	Screeners met participants who initiated the screening. Some participants expressed attraction and curiosity for the screening. Participants were interested in their health and were searching for health solutions. The questionnaire was perceived by the participants as a mark of interest from the screeners. They perceived a benefit from a contact with a caregiver.
			Barriers	Some people did not want to participate. Some expressed they did have no time to undergo the score. Some participants expressed they had other concerns. Some denied the risk they were facing. Some participants expressed to fear their NL-IHRS result. Some participants expressed resistance to change and contemplation. Some alleged they preferred to ignore their cardiovascular status.
**V. Process**				
A	Planning		Facilitators	Organization of the screening was generally satisfactory. Support from junior researchers was appreciated. The planning of screening allowed deployment of screeners in every event. Presentation of the study became fluent. The media coverage was extensive.
			Barriers	Screeners ignored how the project continued after screening. Some unforeseen events appeared during the screening phase: programming difficulties, acceptances to the events were known last-minute. Sometimes, workforce was inadequate to the needs of the events. Screeners underlined that catering could be an issue if no dining area existed or when the provided food was unhealthy compared to cardiovascular disease prevention. Coordination of FP interns could be impaired with lack of anticipation among screeners. A significant time was required from the research team for media coverage.
B	Engaging		Facilitators	None
			Barriers	Expressed barriers to engaging were the organization with students of the preventative health service. They were not involved in the planning construction. The research team provided no feedback to the screeners. The preventative health service was mandatory, so that some students felt forced to participate to the screening.

## 4. Discussion

The efficacy of CVD risk screening in the general population while using mostly health students was excellent. In a 6-month period, 1,309 people at intermediate CVD risk were identified. They will be later invited to the second phase of the SPICES project. The recruitment strategy preserved the FP workforce. One key lesson for the implementation was trust between stakeholders, researchers, and screeners, leading to success. Such a project necessitated repeated, popularized communication, and needed a dedicated position in the research team. Alternative strategies should be planned in case of digital failure or defect, as mobile apps and materials were not totally reliable.

### 4.1. Strengths

This screening in the French general population was the first to be conducted at this scale. This was facilitated by the Interheart risk score. This score was externally validated in seven regions of the world, among which Europe and North America were jointly on one side, and Africa was on another side (5). Evaluating CVD risk with this score enabled mobility of screeners and allowed consideration of unusual sites to perform the screening. Furthermore, the elimination of biological samples, traditionally used when evaluating CVD risk, avoided biological sample management, sampling procedures and logistical issues. Future comparison between every SPICES site is conceivable.

This screening was performed in the general population, which is rare. Patients included in prevention studies are usually recruited in hospitals, clinics, or FP practices, which are not totally representative of the general population. The preventative health service was a new workforce, extremely proactive and efficient for screening activities in the general population and easily reproducible in other countries.

### 4.2. Limitations

#### 4.2.1. Organizational issues

European funding was a major opportunity for the SPICES project. However, as an innovative implementation project, expenditures were unclear when the project was granted. The rigidity of funding lines partially inhibited implementation adaptations. Moreover, the administrative rules for material estimates led to inadequate choices for low-cost unstainable tablets and color-neutral SPICES windbreakers. The research team did not have the final word on choosing between estimates for research material, and the lowest estimate was always selected by the administration, even if it was a poor choice. A better balance between efficacy and cost could improve study results, especially because communication and conciseness of the screening are key components of success. Qualitative data, collected in the CFIR implantation learning climate (Table 3), showed that barriers happened whenever the shortage of research staff prevented the anticipation of project needs. Research teams, when asking for such a grant, should be vigilant when listing their human resource needs.

#### 4.2.2. Selection bias

Although the research team deliberately selected screening sites to be in contact with a varied population and made some efforts to screen in places known to be frequented by men, such as sports events or workplaces, the SPICES cohort was mainly composed of women (63.6%). Performing the screening demanded the participant to be proactive, and women usually use more preventive health care than men (5). Furthermore, the site recruitment strategy was not effective enough to create a representative sample of the COB inhabitants, although this was not the aim of this screening phase. The SPICES screening phase was followed by the second phase of SPICES, which necessitated proactive participants in a 2-year follow-up study.

An unexpected barrier was identified during the screening. Despite a variation in location and type of events, screeners faced a redundant population, leading to decreasing inclusions. As events unfold, screeners met people who were already screened in a previous event. A total of 3,384 assessments were performed among a population of 103,674 inhabitants. It is plausible that screeners had access to the mobile adult population of the territory. The sedentary or poorly socialized population was probably bigger than expected. Getting access to this population would have required other recruitment strategies, such as door-to-door screening. This option was discounted by the research team in order to not overwhelm young students.

#### 4.2.3. Information bias

The NL-IHRS was based on declarative items, and screeners repeatedly reported fearing embellishment from participants, which would lead to information bias. This was not the case, as the score had been previously externally validated in real-life conditions (5). However, the score was validated in some European countries, such as Sweden, Poland, and Turkey (15). These countries are, respectively, classified as moderate, high, and very high CVD risk countries by the European Society of Cardiology, while France is considered a low CVD risk country (16). This could have led to an overestimation of the participants’ cardiovascular risk. No recalibration of the NL-IHRS was known to be performed on a French population.

The NL-IHRS contained no questions about alcohol consumption. Elevated alcohol consumption is known to be a modifiable risk factor (4). Alcoholism is a specific issue of the inhabitants of the studied territory. Premature mortality of men in the COB territory is 45% higher than the French average, alcoholism being the second cause of premature death after suicide (8).

The CVD risk score that is a reference for European countries and France is SCORE 2 (17). SCORE 2 was derived from SCORE in 2021 because of calibration issues among European countries and a decrease in cardiovascular death worldwide (16). SCORE 2 is based on blood tests. It is a reference in medication initiation, especially for cholesterol-lowering drugs. While the overall cohort for validating the SCORE 2 is almost five times bigger than the overall Interheart validation cohort, it involves 599 French individuals solely. These individuals live in Paris and Lyon regions, which have lower CVD death rates than the French average (18). Using the SCORE in the SPICES screening would have probably led to an underestimation of CVD risk, among other issues, such as blood sampling management and delay of results. Instead of identifying unhealthy behaviors, SCORE 2 uses biomedical markers which are only indirectly associated with lifestyle interventions. Therefore, it does not facilitate brief advice to help people adopt healthier ways of life as does the NL-IHRS.

#### 4.2.4. Confusion bias

The NL-IHRS comprised questions on specific dietary habits. Unfavorable habits, according to the NL-IHRS, were infrequent in the cohort. Only 5.5% of participants ate snacks frequently, and 7.9% of participants ate fried foods. These are uncommon food intakes in the territory. Other cultural habits common in the region are the consumption of 3% salted butter, cooking with salted butter and the consumption of processed meats and potatoes. Having observed this NL-IHRS high quality diet, the research team hypothesized that people may have confused potatoes with vegetables instead of starches. The potato status is still under discussion. It has been classified as vegetable by the U.S. and the Australian nutrition guidelines because it is a concentrated source of vitamin C, potassium and contains dietary fibers. Some other food guides, as the French one, exclude potatoes from vegetables because of their association with high-fat diets and their starch content (19). This inaccuracy cannot be currently resolved. Because of regional specificities in food, a cross-cultural adaptation of the dietary items would have been useful and would have likely improved the accuracy of the NL-IHRS.

#### 4.2.5. Strengths and limits of the qualitative interviews

Interviews and coding were performed by junior researchers who knew and had trained the screeners they interviewed. This contributed to an atmosphere of confidence, and no censorship seemed apparent when analyzing the barriers to implementation. This mutual knowledge was perceived by the research team as a strength but from person to person, this could be the opposite. Interviews were conducted after the screening ended for availability reasons. First, the screeners were on annual vacation. Then, the researchers were mobilized at the start of the second phase. This led to a potential recall bias. Interviewing a sample of varied participants who were screened was considered but was not feasible. Some personal data were collected for future participation in the second phase on specific paper lists. Having a parallel list to create a purposive sampling may have risked mixing up the lists for interviews and the second phase.

### 4.3. Comparison to literature

#### 4.3.1. Recruitment for screening

In a 6-month period, 3,384 people were screened. Invitation to the screening could have been performed differently. The DANCANVAS trial aimed to evaluate the efficacy of a cardiovascular screening. The trial used the civil personal registry to recruit men of a Danish region. People of interest were then invited by mail. 62.6% of invited individuals participated (20). Such a procedure is unusual for research purposes in France. Invitation letters are sent for organized breast, colorectal and cervical cancer screenings. Participation to these screenings is, respectively, 55 and 35% for breast cancer and colorectal cancer. For cervical cancer, the current participation is unknown, as this became an organized screening in 2018. Instead of sending mails with unknown efficacy, SPICES chose to be visible on various community places and advertise.

Health prevention centers are other current preventive structures in France from which SPICES could learn. They were created in 1946 and they are supervised by the National Health Insurance Fund for salaried workers. The 85 centers can offer free regular health checks, including cardiovascular health checks, to every worker. Their invitation terms are unspecified. Their activity was recently refocused on precarious people over the age of 16. No efficacy data from these health prevention centers is available, neither for the recruitment nor for the efficacy of the screening (21). The SPICES team could not draw on the experience of these centers.

#### 4.3.2. Comparison of the SPICES population

Mean NL-IHRS for the SPICES cohort was 9.71 (±5.60). France belongs to low risk countries for global cardiovascular risk (16). This consideration is based on few cohort studies. The latest cohort, named EPIC-CVD was used to recalibrate the cardiovascular risk SCORE because of the decreasing rate of CVD related- deaths in Europe. This cohort is exclusively composed of women, which makes it incomparable to this cohort. In the 1980s, the Paris Prospective study concerned exclusively men, and the 5-year incidence of major CVD events was 2.97% (22). Following the NL-IHRS mean score, the 6-year risk of myocardial infarction of this cohort was between 2.1 and 2.4% (5). Although deaths from CVD decreased since the eighties, deaths from cardiovascular diseases are higher in the COB area than the French average.

Tobacco smoking is a major concern in France. Regular daily smokers over 15 years of age are 24% of the total population, according to the European Health Information Gateway (23). The percentage of smokers is 21.6 in the entire UE region. It is 18.9% in our cohort. This difference could be explained by tobacco use of minors (25.1% among 17-years old) and the proportion of women in the cohort, who still smoke less than men.

Hypertension was declared for 18.8% of the cohort. The French cohort ESTEBAN recruited adults aged 18–74 to evaluate the prevalence of hypertension in the French population. The prevalence of hypertension in ESTEBAN was 31.3% (24). Among hypertensive participants, 43.7% were unaware of their condition. It is therefore possible that the SPICES cohort actually includes approximately twice as many hypertensive people.

The French SPICES cohort declared a high-quality diet, which was unexpected. Participants declared a high consumption of vegetables and fruits (respectively, 78.7 and 78.4%), no fried food for 92.1%, no salty food for 94.5% of participants. People were considered having a high consumption of vegetables and fruits if they ate each of these two foods at least once a day. A French study conducted in 2019 found an increase of vegetables and fruits large consumers among the French population. 32% of the population declared eating 5 portions or more per day and 22% declared eating between 2 and 5 servings a day. This increase was partially attributed to a famous French public health message “Eat 5 fruits and vegetables per day,” launched in 2001 (25). Participants may have overestimated their consumption as they knew the expected answer. However, Brittany is the third vegetable producing region in France and France is the fourth producing country in Europe. There is therefore a culture of the consumption of these foods in the COB population.

In the ESTEBAN cohort, diabetes prevalence was 5.7% for diagnosed diabetes. In the SPICES cohort, diabetes prevalence was 4.2%. There is no data available in the COB area to compare this prevalence. More broadly, the prevalence of diabetes in the region is lower than the French average. In 2013, The National Health Insurance estimated this prevalence to be 2.71% in Brittany compared to 3.72% nationally (8).

Using the NL-IHRS in the general population revealed unexpected levels of stress and depression among the general population, although people were screened mostly in their leisure time (40.1 and 24.5%, respectively). Available data on depression and stress among the French population are scarce. In 2017, the prevalence of depression was estimated, using the Composite International Diagnostic Interview-Short Form (CIDI-SF). The prevalence of major depressive episodes in the year was estimated at 9.8% [9.3–10.2%]. This prevalence increased from 2010 to 2017 (26). Earlier, in 2005, the ESEMeD study estimated the prevalence of anxiety disorder at 9.8% and the prevalence of depression at 6.7% using the CIDI (27). The CIDI questionnaires are stricter than the NL-IHRS and allow stress and depression diagnoses to be made in accordance with the DSM IV. However, when creating the Interheart risk score, four questions evaluating stress at work and at home, financial stress, and major life events in the past year were sufficient to identified levels of stress and depression elevating cardiovascular risk (28). The SPICES rates of depression and stress generate cardiovascular health needs for which the current healthcare system is not prepared. Such a level of stress raises the question of systematic stress more than a sum of individual maladjustments.

#### 4.3.3. Barriers and facilitators

The recruitment strategy preserved the FP workforce. One key lesson for the implementation was trust between stakeholders, researchers, and screeners, leading to success. Such a project necessitated repeated, popularized communication and needed a dedicated position in the research team. Alternative strategies should be planned in case of digital failure or defect, as mobile apps and materials were not totally reliable.

A 2022 umbrella review listed barriers and facilitators in health screening (29). The review used a framework to classify barriers and facilitators in five domains as individual factors, social factors, health system factors, healthcare professional and screening procedure factors. This review did not find any previous review addressing specifically cardiovascular prevention. However, common patterns appeared whatever the type of screening was. These findings in these five domains were consistent with the SPICES qualitative data. According to this review, the SPICES screening organization specifically addressed accessibility of screening services. Furthermore, the SPICES screening study provided new and precise information about the project integration, best methods to organize screening in public places and to embed screening in current health professional activities. A difficulty, which was encountered in SPICES, was not reported in the umbrella review. This was the weakness of outdoor digital use and the weaknesses of outdoor digital use and the strategies to be developed to counter them.

### 4.4. Perspectives

The SPICES screening drove a massive number of medical students to a medically deprived area. Health students increased their awareness of preventative health care. Before taking part in the screening, physiotherapist students focused mostly on rehabilitation and physiotherapy treatments. After the screening involvement, new physiotherapists thesis topics appeared, as the role of the physiotherapist in balancing diabetes mellitus. Evidence already existed that training health students specifically to address unhealthy behaviors is effective in reducing cardiovascular risk behaviors (30). However, health students do not feel competent in prevention tasks (31). It is an international issue (32). A quantitative study in 2002 explored Israeli students’ perceptions about their preventive skills in two medical schools. Most of the students’ learning experiences involved hospitalized patients who required treatments. This 2002 study also underlined that the medical schools did not train students in the preventive behavior of patients. Moreover, the French students involved in the SPICES screening were in their third year of training. The first 3 years of medical studies in France are currently focused on basic knowledge and semiology. At the same time, the validation of the Preventative health service is mandatory to continue medical studies. There is a discrepancy between the cursus and competences needed in carrying out prevention. In 2019, a 5-week public health module was developed in New-Zealand, for undergraduate medical students, which could be implemented in France for the Preventative National Health Service. The new course developed an active-based learning approach on both individual-level and population-level case scenarios. Such a curriculum could be integrated in France to improve students’ skills (32). The permanency of the preventative health service in France brought new opportunities of combining teaching, research, and care perspectives, which have been explored in the SPICES screening study.

Because there is still debate about the effectiveness of medications in primary prevention and the burden of medication cost, developing non-pharmacological brief interventions by health professionals is also valuable (33). Having professionals trained in preventative skills early in their careers should improve preventative interactions with patients. As underlined by health professionals, preventative interactions suffer from a lack of financial valuation; there is scope for improvement here. Politicians may have considered spending on prevention merely as an increase in expenditure for their governments. The COVID-19 epidemic raised awareness of EU politicians about benefits that populations could derive from prevention. The EU published new recommendations to invest in both preventative healthcare and preventative social policies. If these recommendations were followed, the challenges raised by cardiovascular prevention could be met (34).

## Data availability statement

The original contributions presented in this study are included in the article/Supplementary material, further inquiries can be directed to the corresponding author.

## Ethics statement

An ethics approval was obtained from the Comité de Protection des Personnes Sud Est IV CPP: 18.12.14.72452; ID-RCB: 2018-A03201-54. The patients/participants provided their written informed consent to participate in this study.

## Author contributions

DL and J-YL participated in the selection process. MG led the scientific committee meeting for the validation of the CVD brief advice. All authors contributed toward data analysis and drafting and revising the manuscript, gave final approval of the version to be published, and agreed to be accountable for all aspects of the work.
