# Supplementary material for: Screening for cardiovascular risk in the general population: The SPICES implementation survey
Source: Front Med (Lausanne). 2023 Jan 16;9:1058090. doi: 10.3389/fmed.2022.1058090 (PMC9885854; doi:10.3389/fmed.2022.1058090)
Supplement: Supplementary file 1 [file Data_Sheet_1.pdf]

Supplementary Material: final versions of semi-structured interview guides by type of population

Supplementary Table 1. Research team final semi structured guide

**Research team final semi structured guide**		
**Theme**	**Open-Ended questions**	**Follow-up probe questions**
Opening question	What were the most exciting moments in this project from your perspective?	
Organization	How were you involved in Spices organization?	Which were the stages of SPICES you were involved in?<br><br>Which parts of the project did you set up?
Barriers and facilitators	What were the local and international barriers and facilitators to SPICES set up?	Which project's requirements were easy or difficult to set up?
Difficulties	Which difficulties did you encounter when you implemented the project?	What were the facilitators to the implementation of the study?
Communication	How efficient was the communication within the team?	How did you communicate locally and internationally?<br><br>What did you appreciate and dislike among your means of communication?

Evolution	How did your level of responsibility evolve during the study?	When were you more involved in the project?<br><br>Are there any tasks you wish you had delegated? If so, which ones and when?
Seclusion	When did you feel secluded to accomplish local or international tasks?	Which organization could have prevented this seclusion according to you?
Excessive workload	When did working for SPICES interfere with your personal life?	Could you share some examples?<br><br>Which organizational solutions, personal or general, would have been adapted?
Areas for improvement	With hindsight, what changes would you have made in your intervention?	What worked best during the project?
Conclusion	Do you remember any highlights we did not share?	

Supplementary Table 2. Preventative health service final semi structured guide

Preventative health service final semi structured guide		
**Theme**	**Questions**	**Follow-up probe questions**
Opening question	What stood out to you the most during the screening?	What is the most striking experience you lived during the screening?
Organization	What is your though about the organization of the screening, before and after the days you screened?	What did you think of the equipment?<br><br>Was the training content appropriate?<br><br>Were the events appropriated to screening?<br><br>What did you think about the media coverage of the project?<br><br>Did people know about the project?
Difficulties	Which difficulties did you encounter?<br><br>A posteriori, which factors are most likely to lead to a failure to perform screening?	The Interheart questionnaire?<br><br>Communication (approach of people, medical knowledge, people's answer)?<br><br>Rejection of screening, refusal to give contact details?<br><br>environment (weather, place, privacy)?<br><br>Uncomfortable situations?<br><br>Off-topic discussions, life stories?

		Which reasons could you imagine for people to refuse the screening?
Facilitators	Which elements helped you in the screening?<br><br>How did you approach people?<br><br>What did you change to screen more people?<br><br>What influences people's acceptance the most, according to you?<br><br>What were the differences between the most and the least effective screeners?	Motivation, welcome of the organizers and the population, health student status, belonging to the project (academic project, involvement of the university hospital, European founding)?<br><br>Solitary screening or group screening?<br><br>Environment?
Self-development	What changed in the way you performed screening between the first and the last person approached?<br><br>How has this experience benefited you personally and professionally?	Any benefit for the current training? For professional future?<br><br>Personal benefit?
Areas for improvement	What are your suggestions for improving such screening?	How should we proceed so that screeners can screen even more people?<br><br>Are there some things to change or to drop because they were useless, tedious, or superfluous?<br><br>Which logistical or material help would be useful to continue this screening (staff, e-tools, advertising items, toolbox…)

Supplementary Table 3. Paramedics final semi structured guide

**Paramedics final semi structured guide**		
**Theme**	**Questions**	**Follow-up probe questions**
Opening question	Did you regret participating to the project even before recruiting one patient?	Why?
SPICES Training	Do you feel the presentation of the SPICES project was clear, and that it appropriately trained you to recruit patients?	Did the junior researcher appropriately present the project at the first telephone interview, and then, at the face-to-face interview?<br><br>Was this presentation enough? Too long?<br><br>How could this presentation be improved to be more efficient?<br><br>Were you prepared enough to the use of the tablet?
Matériel	How did it go with the tablets?	Use by yourself: Were you initially reluctant to use a tablet? Did you encounter technical problems with tablets? How was the screen readability?<br><br>Use by patients: Did patients encounter problems with tablets?
Recruitment period	What did you think about the duration of the recruitment period?	Was it suitable for you and the project? Should have it been longer? Shorter? Why?
Organization	Did the screening and recruitment take up a lot of your professional time?	Was the duration of the screening appropriate to you? Were you reluctant to propose the screening because of the time it would take?

		What was the mean duration of a single screening? Was this acceptable?<br><br>Did the Waist-to-hip ratio take a long time?
Communication	How did the screenings go?	Did you encounter bad or good surprises about your patients? Did you have any difficulties in launching, presenting, and initiating the screening?<br><br>Did you prepare standard arguments beforehand to convince your patients?<br><br>Do you feel a list of arguments, given by the junior researcher, would have helped?<br><br>Did your patients already hear about SPICES?<br><br>What was the first reaction of your patients when you presented them the study?<br><br>Did some includable patients refuse to participate (if yes: lack of time? Discomfort? Pride?)<br><br>Did some patients make remarks about the study or the questionnaire relevance?
Communication	Do you feel your professional status as health professional was helpful to recruit?	Influence of your paramedic status? Familiarity? Authority?<br><br>Was it easier with regular patients?<br><br>Were the patients' reactions helpful or a barrier?

		Influence of the place of screening: was it easier at the patients' home or at the office?
Evolution	Did you change the way you presented the study as you recruited patients?	
Facilitators	What was the most helpful to recruit patients?	
Barriers	What were the biggest difficulties you encountered to recruit patients?	
Sustainability	Do you think screening (in general as specifically for cardiovascular health) by paramedics is sustainable and should be generalized? Why?	
Conclusion	What would you change to improve the screening?	

Supplementary Table 4. Pharmacists' final semi structured guide

**Pharmacists' final semi structured guide**		
**Theme**	**Questions**	**Follow-up probe questions**
Opening question	What motivated you to participate in the SPICES project and to propose the Interheart score?	What are your thoughts about the pharmacist's role in health prevention in general?<br><br>Do you realize other preventive actions?
Organization (initiation)	How did you recruit patients?	What prevented you from proposing screening?<br><br>What did you implement to inform you patients about screening?<br><br>What were the barriers to screening proposal (time, staff...)?<br><br>For what occasion did you realize the screening (every patient, patient coming for prescription renewal…)<br><br>Did the summer period have a specific influence on the screenings?<br><br>Is there a best period in the day to perform screenings?<br><br>Who initiated the screening? If patients, how was he/she aware of the screening (posters, word of mouth, newspapers, physician)? If pharmacists, which arguments did you use to promote the screening?<br><br>If patients were reluctant, which arguments did they provide?

Screening performance	How did you realize the screening?	Where? Duration? By whom?<br><br>Barriers:<br><br>Did you have difficulties to fill the Interheart questionnaire and to perform the waist-to-hip ratio?<br><br>Did you encounter any bug with the tablets? Were they easy to use?<br>Did you feel discomfort in measuring patients?<br><br>Did you have off-topic conversations Off-topic discussions, life stories?
Communication (patients' perceptions)	How did patients react during the interview?	How did they experience the questionnaire?<br><br>Were they comfortable? During the measuring?<br><br>Which kind of follow-up did they want to have?<br><br>Did they think the result matched their experience?<br><br>What were the patients' questions?
Communication	How did you manage the results announcement?	Did you already know the patients' answers?<br><br>How did you manage when patients were includable but refused it?
Self-development	How has this experience benefited you personally and professionally?	

Areas for improvement	What are your suggestions to improve such a cardiovascular screening in pharmacies?	What are your thoughts about integrating pharmacists in a health research project?<br><br>What are the reasons for success of this screening in pharmacies?<br><br>What was missing to improve the number of screenings you performed?
Conclusion	Is there a subject which seems important to you, and we did not cover?	

Supplementary Table 5. FP interns' final semi structured guide

**FP interns' final semi structured guide**		
**Theme**	**Questions**	**Follow-up probe questions**
Opening question	What is the main memory you will keep from this screening experience?	What were the events you participated in?<br><br>What motivated you/ made you want to be part of the SPICES group?
Screening performance	What do you think of the Interheart screening and of the brief advice you gave at the end of the screening?	Do you find it suitable to the cardiovascular risk factors search?<br><br>Do you find it efficient? Easy to deliver? Reproductible?<br><br>How did you feel when you provided the brief advice?

Organization	How did you find the screening organization? Did some elements influence the screening realization?	SPICES stall, supervision<br><br>Screening conditions: type of events, duration of events, weather, solitary realization, group realization
Equipment	How did you manage the equipment provided to perform the screening?	Tablets and software, computer bugs?
Evolution	How did you change the way you performed the screening as you participated to the study?	Revised attitude between the first and the last patient?
Self-development	Would you use the Interheart questionnaire in your daily practice? Why?<br><br>How did screening performance influence you perception of cardiovascular risk factors and their prevention?	According to you, can you expect a personal or professional benefit from that experience?
Areas for improvement	How could such a screening be optimized if it would be repeated in the future?	
Conclusion	Do you remember any highlights we did not share?	
